# Barriers and Enablers to the Production of Open Access Medical Education Platforms: Scoping Review

**DOI:** 10.2196/65306

**Published:** 2025-11-07

**Authors:** Ahmed Abdelfattah Eltomelhussein Ahmed, Arushi Biswas, Nefti Bempong-Ahun, Ines Perić, Eric Patrick O'Flynn

**Affiliations:** 1 Department of Surgical Affairs Royal College of Surgeons in Ireland Dublin Ireland; 2 Johns Hopkins University School of Medicine Baltimore, MD United States; 3 The Global Surgery Foundation Geneva Switzerland; 4 Institute of Global Surgery Royal College of Surgeons in Ireland Dublin Ireland

**Keywords:** open access publishing, medical education, scoping review, educational technology, digital learning, barriers

## Abstract

**Background:**

Free Open Access Medical Education has the potential to democratize access to medical knowledge globally; however, this potential remains largely unrealized, particularly in resource-limited settings. Content is increasingly concentrated on a small number of platforms, each hosting large volumes of material compiled from diverse sources.

**Objective:**

This scoping review aimed to identify and synthesize reported barriers and enablers to the successful design, production, and operation of open access medical education platforms, with the goal of informing strategies to improve their impact, reach, and sustainability.

**Methods:**

We conducted a scoping review using the Arksey and O’Malley framework. A structured search was carried out on April 17, 2023, in PubMed and EBSCOhost. Citation chaining with the SnowGlobe tool and manual reference checking supplemented the search. Studies were eligible for inclusion if they examined platforms that compile content from multiple sources and reported barriers and enablers. Two reviewers (AAEA and AB) independently screened records and extracted data, with discrepancies resolved by a third reviewer (EPOF). Beginning with an a priori framework of “barriers” and “enablers,” coding was then developed inductively. Thematic synthesis categorized findings by stakeholder group.

**Results:**

Of 1108 records identified, 1064 unique records were screened, and 64 full-text papers were assessed; 34 met the inclusion criteria. The most frequently reported barriers were concerns about content-quality control, incomplete or unstructured materials, and the resources needed to sustain platforms long-term. Key enablers included the use of validated tools to assess content quality and collaboration with existing content providers and platforms to enhance visibility and learner engagement. Findings were organized into 3 stakeholder groups: learners and training programs, content designers and creators, and platform managers.

**Conclusions:**

Open access medical education platforms have significant untapped potential to enhance global medical training. Addressing these persistent challenges—particularly around quality assurance, content organization, and sustainability—will require more structured, collaborative, and internationally coordinated approaches.

## Introduction

Advances in technology have revolutionized medical education and training. e-Learning) has become an accepted part of medical training worldwide, at all levels, in both high- and low-income environments [1,2], offering health care professionals and students the promise of accessible and flexible learning opportunities, regardless of their location [3,4]. e-Learning can be defined as “an approach to teaching and learning, representing all or part of the educational model applied, that is based on the use of electronic media and devices as tools for improving access to training, communication and interaction and that facilitates the adoption of new ways of understanding and developing learning” [5]. Included in this definition are a multitude of different forms including asynchronous (self-directed) courses, synchronous (live) tutorials and webinars, blogs, podcasts, social media, learning communities and communities of practice, online videos and images, and more. Learners may interact with this content as part of a formal training program, as formal in-service professional development, or at their own volition. e-Learning content is hosted on a variety of platforms of different types—including learning management systems, content management systems, and learning destination sites. In this review, we will refer to all online locations hosting e-learning content as “platforms.”

Open access content is “digital, online, free of charge, and free of most copyright and licensing restrictions” [6]. This is of particular importance in resource-limited settings, where open access education has the potential to alleviate persistent educational disparities [7]. The acronym “FOAM” (or “FOAMed,” Free Open Access Medical Education) is often used in open access medical education, describing “a dynamic collection of resources and tools for lifelong learning in medicine, as well as a community and an ethos” [8]. As FOAM covers many different types of content, and medical education technologies are quickly evolving, it is difficult to quantify the use of FOAM across the globe and across medical specialties. However, it is clear that there is a lot of FOAM content available, that it is increasingly accepted as a valid medium for medical education, and that it is increasingly used. Taking podcasts as one example, a study looking at podcasts related to a limited set of medical specialties found 169 different English language podcasts with over 6500 combined hours of educational content [9]. Acceptance of podcasts as a medical education tool is growing. While a 2007 study found that most of the US students and physicians surveyed felt podcasts had no role in medical education [10], a 2019 study reported that 71% of residents from various US training programs supported the utility of podcasts [11]. In total, 39% of US nephrology fellows report regularly learning from podcasts [12].

Open access does not, however, mean equal access. Use is higher in high-income countries than in low- and middle-income countries (LMICs) [13], and LMIC learners face additional challenges [14]. A systematic review of e-learning for medical education in LMICs finds that it has “not met its expected potential” in such settings [15]. Even within a single LMIC institution, some learners have greater access than others [16]. Thus, due to these disparities, open access platforms may not in fact fulfill their promise of decreasing pre-existing educational [17], health care, and economic inequalities [18]. This is a profound issue: those most in need of medical education and training information, such as professionals in resource-limited settings, often find it the most elusive [19].

The total number of FOAM sites now seems to be decreasing, as open access medical education resources are increasingly consolidated in a smaller number of platforms [20-22]. Due to the effort and expense involved in producing, maintaining, and hosting e-learning content, it can be expected that this trend will continue; the number of platforms will decrease, yet the volume of content on each platform will increase, compiled from multiple sources. Barriers and enablers to the production and sustainability of e-learning material of various kinds have been described in the literature [23]. This review aims, for the first time, to identify and synthesize from the literature the barriers and enablers to the successful design, production, and operation of e-learning platforms compiling content from multiple sources. In doing so, this review looks to inform efforts to optimize the impact and reach of open access medical education resources.

## Methods

### Study Design

A scoping review methodology was selected “to ‘map’ relevant literature in the field of interest,” following the framework proposed by Arksey and O’Malley [24]. The protocol was publicly registered on Open Science Framework [25]. Results are reported in line with the PRISMA-ScR (Preferred Reporting Items for Systematic Reviews and Meta-Analyses extension for Scoping Reviews) [26]. The PRISMA-ScR checklist is included as Multimedia Appendix 1.

### Research Question

We defined our research question as “What is known in the existing literature about the barriers and corresponding enablers to developing, producing, and maintaining open access medical education platforms containing material from multiple sources?”

### Search Strategy

Searches were conducted on April 17, 2023, using PubMed and EBSCOhost, which includes Academic Search Complete, ERIC, and CINAHL Plus with Full text. The search string used was ((resources) OR (material)) AND ((((medical education) OR (training)) AND (((((identify) OR (evaluate)) OR (integrate)) OR (compile)) OR (collect))) AND (open-access)). The SnowGlobe tool [27] was then used for citation chaining (snowballing), automatically retrieving references of included studies as well as studies citing them. Manual reference searching additionally contributed a number of papers. Search results were imported into Covidence (Veritas Health Innovation), and duplicates were removed. Gray literature, such as institutional websites, preprints, and reports, was not searched.

### Study Selection

Studies were eligible for inclusion if they described open access medical education or training platforms that curate or compile content from multiple sources or institutions for use by health care providers. To be included, studies also needed to provide information on factors that facilitate or hinder the design, production, implementation, or ongoing operation of such platforms. All paper types, including reviews, were considered. No date restrictions were applied, given the emerging nature of this field. Studies were excluded if they focused solely on a single course or on materials developed by a single institution. Conference abstracts and non-English language studies were also excluded.

All identified papers underwent a 2-stage screening process. In stage 1, a total of 2 authors (AAEA and AB) independently reviewed the title and abstract of each paper against the predefined inclusion and exclusion criteria. Discrepancies were resolved with the input of EPOF. In stage 2, the full texts of potentially relevant papers were assessed for final inclusion by AAEA and AB, with EPOF adjudicating on discrepancies.

### Charting the Data

Beginning with an a priori framework of “barriers” and “enablers,” the data chart form was then developed through an inductive process, with input from all authors. Following initial immersion in the data, coding for each barrier and enabler was developed. Data from included papers were then independently extracted by AAEA and AB into this form. Any disagreements were resolved through discussion or with the input of EPOF. Finally, the coding for barriers and enablers was reviewed, and minor coding edits were made. To derive practical recommendations for the various stakeholder groups involved in the production, management, and use of open access educational platforms, barriers and enablers were then mapped to the appropriate stakeholder group: (1) learners and training programs, (2) content designers and creators, and (3) platform managers.

### Data Synthesis and Reporting

Thematic analysis was undertaken. Once authors were familiar with the data, and coding agreed, themes were then proposed, explained, and clarified through iterative team discussion. Both barriers and enablers were then grouped under these themes, and the themes were reviewed for appropriateness. Descriptive statistics were used to analyze characteristics of the included studies.

### Ethical Considerations

The review involved analysis of publicly available secondary data only. No human or animal participants were involved; therefore, ethics approval was not required.

## Results

### Overview

Our search strategy identified a total of 1108 records from multiple sources. PubMed contributed 502 records, followed by 253 from EBSCOhost databases (including Academic Search Complete, ERIC, and CINAHL Plus with Full Text). Snowball sampling added 332 records, and 21 were identified through manual reference searching. After removing duplicates, 1064 records were screened by title and abstract. Of these, 64 full-text papers were assessed for eligibility.

**Figure 1:**
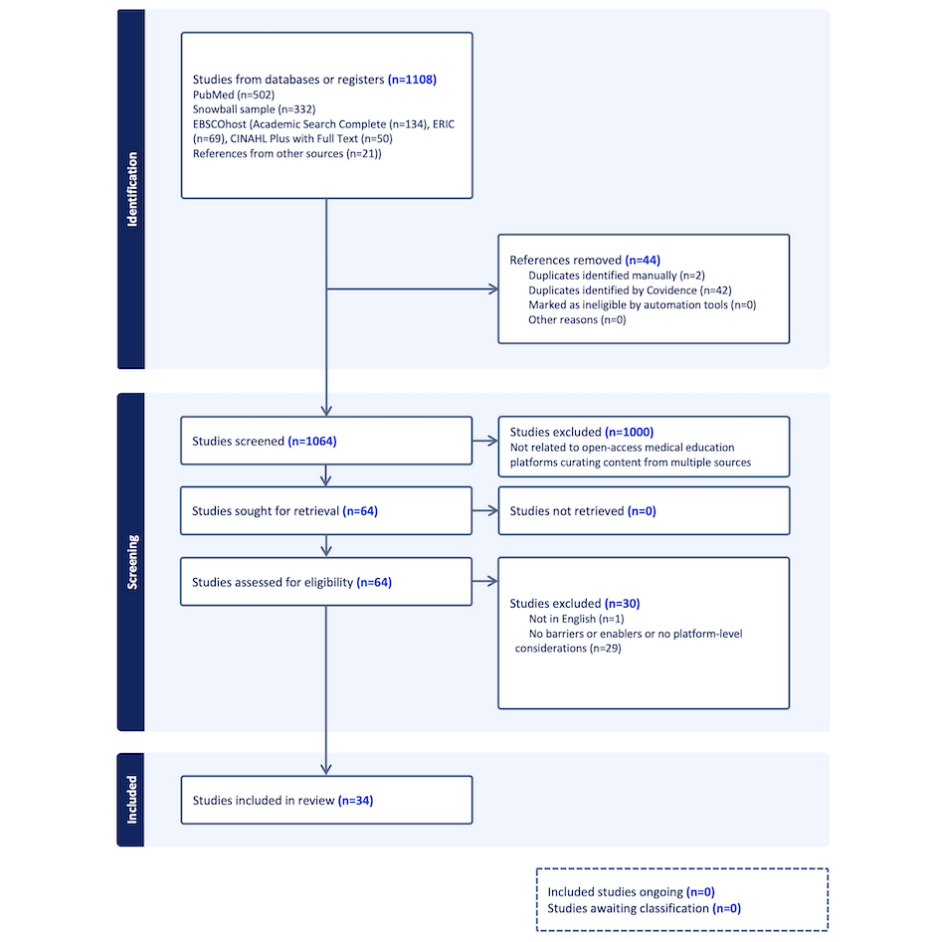
PRISMA (Preferred Reporting Items for Systematic Reviews and Meta-Analyses) flow diagram.

A total of 29 full-text studies were excluded because, although they addressed open access content, they did not examine barriers or enablers or did not relate to platform-level considerations. Ultimately, 34 studies were included in the final review. The study selection process is shown in Figure 1.

### Characteristics of the Included Studies

All 34 studies described educational resources targeted at doctors or medical students. Nurses were also targeted in 14 (41%) studies, and allied health professionals in 11 (32%) studies. Most studies did not explicitly focus on a particular geography. Some focused on LMICs [2,28], while others were explicitly “global” [29] or “international” [30] in their scope. However, most first authors were based in high-income countries, with a majority from the United States. Other first authors were from Canada, Germany, Belgium, Italy, the United Arab Emirates, the United Kingdom, Australia, Botswana, Papua New Guinea, and South Africa. In total, 16 (47%) studies were categorized as reviews or expert opinion, 11 (32%) were primarily qualitative research, 5 (15%) were primarily quantitative research, and 2 (6%) were reports on educational innovations. While all included studies discussed open access medical education platforms, this was rarely their primary focus. Most included studies focused on e-learning in medical education in general, or in specific medical education and training contexts, or specific topics and specialties, or for specific cohorts of learners. Other studies again focused on the FOAM education movement. Characteristics of the included studies are given in Table 1.

**Table 1:** Characteristics of included studies.

Author name (year)	Country of first author	Type of study	Platform or context	Barriers	Enablers
Kleinpell et al (2011) [31]	United States	Qualitative research	Early open online nursing education resources (blogs, wikis, repositories); emphasis on discoverability and maintenance burdens.	Navigability, searchability Access Effort and cost to maintain	Crowdsourced feedback Interactive content Tools to help learners find resources Student-centered, personalized Content integrated with curricula Networking of educators Tools to make content creation easier
Nieder et al (2022) [2]	Germany	Scoping review	Global or LMIC^a^-friendly open resources for medical education (FOAM^b^-style blogs or podcasts, YouTube, mobile-first sites) with attention to bandwidth and language.	Computer, phone, internet Time Language Access Digital literacy Learning culture	Collaboration for awareness Social, instructor, and cognitive presence Learner familiarity Low bandwidth content Local language External pressure Appropriate content structure Interactive content Quality tool Regular content review
Pizzolato et al (2020) [32]	Belgium	Qualitative study	Open educational websites with structured navigation and mapped content areas to guide learners.	Incompleteness, lack of structure Interactivity, design	Navigable, searchable Interactive content Map content areas
Grock et al (2019) [33]	United States	Qualitative research	FOAM resources in emergency medicine; blogs or podcasts and their quality or appropriateness concerns.	Volume of content Quality control Appropriateness of content Incompleteness, lack of structure	Quality tool
Schettino and Capone (2022) [34]	Italy	Scoping review	Open platforms used in undergraduate medical curricula; focus on training content creators and interactive, learner-centered design.	Language Digital literacy Learning culture Motivation Time	Local language Student-centered, personalized Appropriate content structure Training of content creators Interactive content
Bansal (2021) [35]	Australia	Expert opinion	Broad FOAM ecosystem across blogs, podcasts, Twitter, YouTube, and online question banks; strategies to expand reach and impact.	Navigability, searchability Interactivity, design Quality control Motivation Learning culture Effort and cost to maintain	Collaboration for awareness Networking of educators Training of content creators
Regmi and Jones (2020) [23]	United Kingdom	Systematic review	Systematic review of digital or open resources integrated with trainee experience and formal curricula; clarity and structure emphasized.	Digital literacy Motivation Interactivity, design Appropriateness of content Incompleteness, lack of structure Effort and cost to build Effort and cost to maintain	Content integrated with trainee experience Content integrated with curricula Clarity of content Student-centered, personalized Appropriate content structure Interactive content
Culbert et al (2022) [36]	United States	Review paper	Review of open access educational resources in medicine; integration into curricula and institutional adoption.	Access Quality control Quality tool issues Effort and cost to maintain	Collaboration for awareness Content integrated with curricula
Rodman et al (2021) [28]	United States	Needs assessment	Needs assessment for OERs^c^, preference for locally authored content, and regular review cycles.	Language Effort and cost to maintain Appropriateness of content	Technological advancement Local language Local content creators Regular content review
Cevik et al (2021) [30]	United Arab Emirates	Expert opinion	Expert guidance on leveraging FOAM or OER with curation and Creative Commons licensing; curated compilations to aid discovery.	Computer, phone, internet Awareness Language Interactivity, design	Collaboration for awareness Curated compilations Open Access and Creative Commons
Knopf et al (2020) [37]	United States	Descriptive analytical study	Descriptive analysis of learner engagement with open platforms; emphasis on social or instructor presence and tool quality.	Computer, phone, internet Language Learning culture Quality control Incompleteness, lack of structure	Social, instructor, and cognitive presence Quality tool
Chan et al (2019) [38]	Canada	Expert opinion	Critical appraisal of FOAM resources; encourages structured evaluation frameworks for blogs or podcasts and social media posts.	Volume of content Trust Quality control Incompleteness, lack of structure	Learner familiarity Collaboration for awareness Peer review, editorial process Quality tool Crowdsourced feedback Map content areas
Cameron and Schofield (2017) [29]	Canada	Review paper	Review of open online medical education with emphasis on ethics, intellectual property, and learner-centered design; recommends low-bandwidth delivery.	Computer, phone, internet Learning culture Motivation Effort and cost to build Ethics Intellectual property	Low bandwidth content Content integrated with trainee experience Clarity of content Student-centered, personalized Appropriate content structure Immediate feedback Social, instructor, and cognitive presence Existing ethical guidelines Open Access and Creative Commons
Lin et al (2016) [39]	United States	Educational innovation report	Approved Instructional Resources initiative; curated, peer-reviewed emergency medicine FOAM blogs or podcasts aligned to curricula.	Quality control Quality tool issues Incompleteness, lack of structure	Collaboration for awareness Curated compilations Free tools for hosting content
Thurtle et al (2016) [40]	Australia	Qualitative research	Qualitative exploration of FOAM awareness and trust; learners relying on local creators and site familiarity.	Computer, phone, internet Awareness Trust Navigability, searchability	Learner familiarity Local content creators
Thoma et al (2015) [41]	Canada	Educational innovation report	Implementation of peer review on a major FOAM blog to improve quality assurance and transparency.	Expertise of authors Quality control	—^d^
Thoma et al (2014) [42]	Canada	Perspective paper	Perspective on navigating FOAM: curated compilations, mentor-recommended resources, and residency-endorsed lists.	Trust Navigability, searchability Volume of content Quality control	Tools to help learners find resources Resources recommended by colleagues, mentors Curated compilations Resources recommended by training programs
Alexiou and Falagas (2008) [43]	Greece	Expert opinion	Early expert commentary on open online resources in medical education; calls for better organization and curation.	Volume of content Incompleteness, lack of structure Effort and cost to maintain	Tools to help learners find resources Interactive content Curated compilations
Grock et al (2021) [44]	United States	Observational study	Analysis of FOAM coverage of core curricular topics; identifies gaps relative to competency frameworks.	Navigability, searchability Quality control Incompleteness, lack of structure	—
Grock et al (2021) [45]	United States	Usability study	Revised Approved Instructional Resources initiative; curated, peer-reviewed emergency medicine FOAM blogs or podcasts aligned to curricula.	Quality control Quality tool issues	Quality tool
Ghiathi et al (2020) [46]	United States	Evaluation study	Evaluation of engagement strategies and tooling that make creating and maintaining open content easier.	Quality control Quality tool issues	Collaboration for awareness Engagement strategy Tools to make content creation easier
Misra and Lawson (2019) [47]	United States	Technical report	Technical report on operationalizing open resources; stresses maintenance effort and need for periodic review.	Incompleteness, lack of structure Effort and cost to maintain	Quality tool Curated compilations Regular content review
Hsiao et al (2021) [48]	United States	Review paper	Review of topic-focused open resources; supports curated lists and quality tools for better navigation.	Navigability, searchability Volume of content Quality control Incompleteness, lack of structure	Quality tool Curated compilations
Lin et al (2022) [21]	United States	Cross-sectional study	Census of active emergency medicine or critical care FOAM blogs or podcasts; sustainability and trend analysis.	Dated content Effort and cost to build Effort and cost to maintain	Collaboration for awareness Content integrated with curricula
Mncube and Mthethwa (2022) [49]	South Africa	Qualitative research	Qualitative study of FOAM in South Africa; emphasizes open licensing and social or instructor presence.	Awareness Access Quality control Effort and cost to maintain Ethics	Collaboration for awareness Social, instructor, and cognitive presence Open Access and Creative Commons
Zhang et al (2019) [50]	Canada	Observational study	Observational work on open platform structure and interactivity; highlights the need for quality tools.	Navigability, searchability Interactivity, design Quality control Incompleteness, lack of structure Effort and cost to build	Collaboration for awareness Technological advancement Appropriate content structure Interactive content Quality tool
Grabow Moore et al (2021) [51]	United States	Descriptive study	Descriptive account of learner-centered open content design and routine review processes.	Computer, phone, internet Time Quality control Incompleteness, lack of structure Effort and cost to maintain	Student-centered content design Crowdsourced feedback Regular content review
Lee et al (2022) [52]	Canada	Economic evaluation	Economic evaluation of creating or maintaining OERs; discusses funding or recognition mechanisms.	Effort and cost to build Effort and cost to maintain	Learner familiarity Funding and recognition for content creation
Wolbrink et al (2019) [53]	United States	Analytic review	Review of selected open resources for critical care.	Access Interactivity, design	Navigable, searchable Appropriate content structure Interactive content Peer review, editorial process Quality tool Regular content review Map content areas
Shappell et al (2017) [54]	United States	Review paper	Review of FOAM ecosystems; maps content areas, curated compilations, and crowdsourced feedback models.	Interactivity, design Quality control Incompleteness, lack of structure	Collaboration for awareness Appropriate content structure Interactive content Quality tool Crowdsourced feedback Curated compilations Map content areas
Evans et al (2022) [55]	United States	Cross-sectional study	Cross-sectional study of open resources, processes, and tooling (peer review, quality instruments) across sites.	Computer, phone, internet Access Interactivity, design Quality control Quality tool issues Effort and cost to build Effort and cost to maintain	Collaboration for awareness Interactive content Peer review, editorial process Quality tool
Lo et al (2018) [56]	Canada	Expert opinion	Expert commentary on integrating FOAM into formal education; highlights peer-reviewed tools and mentor-curated lists.	Volume of content	Resources recommended by colleagues, mentors Interactive content Quality tool
Ting et al (2020) [57]	Canada	Literature review	Quality appraisal or assurance techniques for FOAM; proposes methods to evaluate blogs or podcasts and social media content.	Ethics Quality control Ethics	Content integrated with curricula Quality tool Funding and recognition for content creation
Marée (2019) [58]	Belgium	Mini review paper	Mini review on OER or FOAM with focus on maintenance costs and intellectual property; advocates collaboration for awareness.	Computer, phone, internet Incompleteness, lack of structure Effort and cost to maintain Intellectual property	Collaboration for awareness

^a^LMIC: low- and middle-income country.

^b^FOAM: Free Open Access Medical Education.

^c^OER: open educational resource.

^d^Not available.

Across the 34 included studies, we identified 127 instances of barriers and 122 instances of enablers. These were coded into 22 unique barriers and 33 unique enablers, and then, mapped to 1 of 3 stakeholder-based themes. A full list of the coded barriers and enablers, and the frequency with which they were identified, is presented in Tables 2 and 3.

**Table 2:** Frequency and detail of barriers identified.

Barrier name			Barrier detail		Values, n
**Theme: learners and training programs**					
	Learning culture	Lack of learner culture of e-learning, and lack of culture of or dislike of interactive learning		5	
	Motivation	General lack of motivation and adherence		4	
	Digital literacy	Lack of learner digital literacy		3	
	Time	Learners’ limited time availability or conflict of priorities		3	
	Trust	Learners’ lack of trust in content, lack of connection to content creators		3	
**Theme: content designers and creators**					
	Quality control	Lack of quality control		18	
	Incompleteness, lack of structure	Material does not equally cover all required learner knowledge areas and is not presented in a logical structure		14	
	Interactivity, design	Lack of interactivity of resources and poor design		8	
	Computer, phone, internet	Learners’ lack of access to adequate computer, phone, or internet		8	
	Navigability, searchability	Resources are difficult to find and navigate		7	
	Volume of content	Overwhelming volume of content		6	
	Language	Lack of learner competence or comfort in the language of resources		5	
	Quality tool issues	Issues with quality tools used		5	
	Appropriateness of content	Content not appropriate for learners’ context and level		3	
	Dated content	Dated content		1	
	Expertise of authors	Lack of author subject area expertise		1	
**Theme: platform managers**					
	Effort and cost to maintain	Human and financial resources required to maintain and update content		13	
	Access	Restrictions on learner access to material (requirement to register or be a member of certain organizations)		6	
	Effort and cost to build	Human and financial resources required to develop content		6	
	Awareness	Learners’ lack of awareness of resources available		3	
	Ethics	Ethical issues		3	
	Intellectual property	Intellectual property issues		2	

**Table 3:** Frequency and detail of enablers identified.

Enabler name			Enabler detail		Values, n
**Theme: learners and training programs**					
	Curated compilations	Use of curated compilations of resources		7	
	Learner familiarity	Growth of learner familiarity with and awareness of e-learning and FOAM^a^ in general		4	
	Resources recommended by colleagues and mentors	Recommendations of resources by trusted colleagues and mentors		2	
	External pressure	External circumstances and pressures		1	
	Engagement strategy	Production of a learner engagement strategy		1	
	Resources recommended by training programs	Curation or recommendation of resources by training programs		1	
**Theme: content designers and creators**					
	Quality tool	Use of a tool to rate quality of online resources or framework development		13	
	Interactive content	Interactive content		11	
	Appropriate content structure	Appropriate content structure (eg, iterative, with learning objectives, using multimedia)		7	
	Content integrated with curricula	Integration of resources with established curricula		5	
	Regular content review	Regular content review		5	
	Student-centered, personalized	Student-centered, personalized learning approach		5	
	Crowdsourced feedback	Crowdsourced feedback and curriculum development		4	
	Social, instructor, and cognitive presence	Establishment of a social presence (peers), instructor presence, and cognitive presence (material and assignments)		4	
	Map content areas	Mapping and categorization of content areas, aiming for comprehensiveness		4	
	Local language	Content translation into local language		3	
	Peer review, editorial process	Peer review, editorial process		3	
	Navigable, searchable	Systematic characterization of resources for navigability and searchability		2	
	Networking of educators	Networking of educators, sharing best practice		2	
	Training of content creators	Training of content creators		2	
	Clarity of content	Clarity of content		2	
	Content integrated with trainee experience	Integration of content with trainee experience		2	
	Local content creators	Recruitment of content creators from the same context as learners or launch LMIC^b^ platform		2	
	Technological advancement	General global technological advancement		2	
	Low bandwidth content	Use of low-bandwidth content where the internet is poor or expensive		2	
	Immediate feedback	Provision of immediate feedback to learner		1	
**Theme: platform managers**					
	Collaboration for awareness	Collaborate and partner with existing content providers or hosts to increase awareness		13	
	Tools to help learners find resources	Tools to help learners find resources (eg, automatically queued resources and custom search engines)		3	
	Open Access and Creative Commons	Availability of Open Access and Creative Commons resources		3	
	Funding and recognition for content creation	Funding and recognition for content creation		2	
	Tools to make content creation easier	Use of tools to make content creation easier		2	
	Free tools for hosting content	Use of free tools for hosting content		1	
	Existing ethical guidelines	Use of existing ethical guidelines		1	

^a^FOAM: Free Open Access Medical Education.

^b^LMIC: low- and middle-income country.

### Learners and Training Programs

#### Barriers

Most barriers under this theme related to learner engagement: the lack of an interactive learning culture conducive to self-directed e-learning, particularly in resource-limited settings [2,29,34,35,37]; a lack of trust in content or content creators [38,40,41]; and a general lack of learner motivation and adherence [23,29,34,35]. Practical barriers were a lack of learner digital literacy [2,23,34] and learners’ limited available time due to competing priorities [2,34,51]. Barriers under this theme were particularly reported by studies in resource-limited contexts.

#### Enablers

Some of the barriers found can be expected to be partially addressed by global macrotrends, such as increasing learner familiarity with, and acceptance of, e-learning and interactive learning approaches [2,38,40,52]. Several enablers were reported, which may address challenges of learner trust and motivation—peer-to-peer recommendation of content [42,56], training bodies recommending specific content to their trainees [42], and the use of trusted curated compilations of resources [30,39,42,43,47,48,54]. Other enablers can be leveraged by training programs to increase engagement, such as the development of a formal engagement strategy [46]. External pressure, such as when completion of certain e-learning content is determined to be a mandatory part of a training program, with appropriate deadlines and reminders, was also reported as an enabler [2].

### Content Designers and Creators

#### Barriers

Barriers reported relating to content creation and design included a lack of resource interactivity [23,30,32,35,50,53-55], resources that are difficult to find and navigate [31,35,40,42,44,48,50], and an overwhelming volume of content [33,38,42,43,48,56]. However, the major challenge under this theme—and the most cited barrier in this study—was a lack of quality control, which was identified in the majority of included studies (n=18, 53%) [33,35-39,41,42,44-46,48-51,54,55,57]. Other quality-related barriers reported were problems with quality evaluation tools [36,39,45,46,55], a lack of author subject area expertise [41], and dated content [21].

Incompleteness of content or lack of structure—where material does not appropriately cover all required learner knowledge areas or is not presented in a logical structure—was repeatedly reported [23,32,33,37-39,43,44,47,48,50,51,54,58]. Some topics appear to be covered in detail on multiple platforms, while other topics are seemingly absent. Grock et al [44] describe the “uneven distribution” of open access content as “holes in the FOAM.” Content in some cases was also reported not to be at the appropriate level for target learners or not appropriate for the learners’ context [23,28,33].

Finally, practical barriers found that can be taken into consideration by content designers and creators were learners’ lack of access to adequate equipment and infrastructure such as computers, phones, and reliable internet connections [2,29,30,37,40,51,55,58] and lack of learner competence in the language in which content was provided [2,28,30,34,37].

#### Enablers

Many of the enablers reported under this theme can be considered general good practice in adult education, such as interactive learning approaches [2,23,31,32,34,43,50,53-56], clarity of content [23,29], the provision of immediate feedback to the learner [29], and student- or learner-centered design [23,29,31,34,51]. The “flipped classroom” was cited as an example of “an interactive learner-centric approach particularly well-suited to the needs of contemporary ... learners” [51]. Gamification was reported as another effective student-centered design approach, as it “represents a way to increase the attractiveness of learning content among students and foster their motivation to participate in the proposed activities” [34]. Other enablers centered on e-learning design, such as the use of an appropriate structure with learning objectives, multimedia, and the employment of an iterative design process [2,23,29,34,50,53,54]. Another reported enabler was the establishment of 3 forms of “presence”: social presence (peers), instructor presence, and cognitive presence (material and assignments) [2,29,37,49].

Two enablers reported focused on the development of content creators: the networking and sharing of best practices among educators [31,35] and the training of content creators [34,35]. Bansal [35] proposes faculty development programs to “train a cohort of clinical educators who can drive the creation of FOAM resources.” The final enabler under this theme was the systematic characterization of resources for navigability and searchability [32,53].

The most commonly reported enabler to address the issues of content quality was the use of objective, validated content quality evaluation tools to assess the quality of content before publication [2,33,37,38,45,47,48,50,53-57]. Examples given include the “Approved Instructional Resources” tool [45] and the revised “Medical Education Translational Resources: Impact and Quality” tool [59]. Other enablers addressing issues of content quality were the implementation of regular content review [2,28,47,51,53], content peer review as part of a transparent selection and evaluation process [38,53,55], and crowdsourced feedback [31,38,51,54].

Two enablers under this theme focused on ensuring that content was appropriate for the target learner’s level and context: the recruitment of content creators from the same context as targeted learners [28,40] and the integration of learners’ experience into content [23,29]. Integration of resources with established curricula [21,23,31,36,57] was reported as a means to ensure both content appropriateness and comprehensiveness. Mapping and categorizing content areas [32,38,53,54] were reported as a way to identify content area gaps and thus enable the production of more complete and comprehensive learning resources.

Where the internet connection of the target learner population is likely to be expensive or of poor quality, ensuring content is not bandwidth-intensive was an identified enabler [2,29]. In such contexts, Nieder et al [2] propose “[p]roviding access to course media such as video content in lower resolution and downloading course materials for offline usage in places with a better connection.” The global spread of technology and improvements in internet speed can also be expected to enable greater access over time [28,50]. Learner participation was reported to be facilitated by translation of content into local languages [2,28,34].

### Platform Managers

#### Barriers

Barriers found, which can be addressed by platform managers, include learners’ inability to access content due to requirements to register or be a member of an organization [2,31,36,49,53,55]. Lack of learner awareness of available resources [30,40,49] can also be addressed by platform managers.

A number of barriers to the long-term sustainability of open access platforms were reported—most commonly, the resources and efforts required to maintain and update content [21,23,28,31,35,36,43,47,49,51,52,55,58]. Lin et al [21] note that open access medical education platforms originated from “volunteers providing free education to all who wish to learn. This volunteerism comes at the expense of opportunity costs and may have been unsustainable for many sites that no longer exist.” The direct costs of content development are also a notable barrier [21,23,29,50,52,55]. Barriers that were reported related to intellectual property [29,58], included questions over the legality of the common practice of reproducing content from journal papers [29]. Ethical concerns, such as the deliberate sharing of incorrect information, bullying on social learning spaces, and the unethical appropriation of material, were also raised [29,49,57].

#### Enablers

Collaboration between content providers and platforms was repeatedly reported as an enabler, in order to make resources available on highly visible platforms, thus addressing a lack of learner awareness of these resources [2,21,30,35,36,38,39,46,49,50,54,55,58]. Several relatively simple technological enablers were also found. Where cost is a barrier, the use of free tools [39], such as Google Drive for content hosting, was reported as an enabler. User-friendly content creation tools were reported to reduce the effort required to produce resources [31,46]. Incorporation of tools into platforms, such as custom search engines to help learners find resources [31,42,43], was proposed to address barriers around awareness, searchability, and navigability.

Financial support and academic recognition for content creation were seen to enable the long-term effort required of content creators [52,57]. The use of Creative Commons licenses to clarify the legal status of content was reported as an enabler to address intellectual property–related barriers [29,30,49]. Users are free to retain, reuse, revise, remix, and redistribute the content (the 5Rs) under Creative Commons licenses, which have a range of openness depending on how many of the 5Rs are allowed to the user. Mncube and Mthethwa [49] consider that “Once such [Open Access Educational Resource] common laws are well established in faculty ... collaboration ... will continue to grow unviolated.” One study reported the use of existing ethical guidelines as an enabler [29].

## Discussion

### Principal Findings

As has already been seen in the specialties of emergency medicine and critical care [21], it seems likely that open access medical education resources in other areas and specialties are becoming increasingly consolidated in a smaller number of larger online platforms. Such compilations of open access medical resources have the potential to improve the learner experience by offering a “1-stop shop,” where learners are confident that they can find easy-to-use, quality-assured, up-to-date, comprehensive learning resources appropriate for their context.

Our review finds numerous barriers reported to the design, production, and operation of such platforms. The literature also shows that there is much that open access content designers, authors, and platform administrators can do to address these barriers; and indeed, there is much that can be done by training programs and learners themselves. We can reasonably expect that some of these barriers will diminish in severity over time. Some of the practical barriers identified, particularly in resource-limited settings [2], will partially be addressed by improving internet connectivity, while growing learner familiarity with interactive online learning methodologies can be expected to partially address some of the cultural and motivational barriers.

The 3 most cited barriers in this review were a perceived lack of content quality control, incompleteness of content, and concerns that the demand on human and financial resources required to maintain and update content are unsustainable.

While informal peer and expert recommendations of resources may be helpful, neither learners nor experts are consistently able to make an accurate “gut feeling” appraisal of the quality of online educational resources [60]; therefore, informal recommendation of resources alone may not suffice as a quality control measure. Objective, validated content quality evaluation tools such as the “Approved Instructional Resources” tool [45] or the revised “Medical Education Translational Resources: Impact and Quality” tool [59] should be routinely used by content creators and open access platform curators.

Where e-learning content is targeted at students or trainees in formal training programs, such content should be integrated with learners’ other educational activities, rather than existing apart from them. The connection between content and curricula should be made clear and explicit through a mapping of content to appropriate established curricula. Training programs should identify appropriate open access resources, which fulfill the requirements of their curricula and should guide their students and trainees toward them. The curation of content into platforms, and the linking of content and curricula, will make clear which training needs are met and which are not met by currently available open access content. Identification of content area gaps will reduce duplication of effort and allow “holes” [44] in open access medical education content to be filled by appropriate content.

The barrier presented by the human and financial resources required to maintain and update content is the least amenable to the solutions found in this review. Enablers addressing the sustainability of human resources, such as paying content creators, may negatively impact the financial sustainability of such platforms. Cost and effort may be contained to some degree by the enablers identified in this review; however, creating, hosting, and updating high-quality content will continue to cost money and require significant effort. Hosting content on curated platforms, such as the United Nations Global Surgery Learning Hub [61] and OpenCriticalCare [62], which are free for both content providers and learners, may address some of the issues associated with long-term hosting of content as well as partially address issues of quality control and trust. Ultimately, the value of material and platforms will have to be acknowledged for the associated costs to be met.

### Comparison With Prior Work

This review differs from prior published work by focusing on open access e-learning platforms compiling content from multiple sources, whereas previously published literature has predominantly focused on specific courses and cohorts. Many of the barriers and enablers identified relating to platforms have previously been found in studies of individual courses, and meta-analyses relating to e-learning in health sciences in general [23] and e-learning in medical education in LMICs [15].

These include learner motivation and familiarity with e-learning, incompleteness and inappropriateness of content, and challenges in maintaining and updating content. In LMICs, basic challenges in getting online were highlighted [15]. Enablers related to all of these barriers were similar to those found in this study. Some challenges related to courses are not applicable to platforms. Barteit et al [15] found a preponderance of pilot or small-scale e-learning interventions for resource-limited settings that never produced content at scale. De facto compilations of medical education contain significant volumes of content.

This study found the financial sustainability of open access medical education compilations, and the sustainability of the level of effort required, to be challenges with few straightforward solutions. This concern is echoed throughout the broader literature on open access medical education. Lee et al [52] calculated the median overall value of a FOAM website to be US $22,815 and concluded that there is “substantial value being generated from these resources and ... this should be recognized by academia.” Platform costs will either have to be met by funding from academic institutions and funding bodies or by charging fees—to content providers, to learners in high-income settings, to training programs, or for learners to access “premium” content. Lin et al [21] found that the majority of the 109 FOAM sites that they analyzed generated income, with 18% embedding advertisements; 27.5% asking for donations or payment for merchandise, continuing medical education credits, books, or web-based courses; and 44% being affiliated with a sponsoring institution, such as a professional society, hospital, or journal. Lee et al [52] draw parallels between the FOAM movement and the open access publication movement, noting that in making publications available open access, publishers shifted the cost from the content consumer to the content producer. They note that grant funding mechanisms exist to fund these publication costs and call for similar support for medical education content creation. Mncube and Mthethwa [49] quote an unnamed academic, “When we write and publish the research articles or book chapters we are incentivized, however in the development of [open access resources], there are no incentives.” Where it is not possible to fund content creators, and until more sustainable models are developed, greater acknowledgment and recognition of content creators and others involved in this work may be a simple way to help sustain their motivation.

Two important barriers found relating to compilations of medical education resources, that were either not discussed or not given prominence in the literature on individual courses, are issues of trust and language. It may simply be that analyses at a course level exclude potential learners who either do not trust the content or are not comfortable learning in the course language of instruction—they would never take the courses in the first place. We found that managers of such platforms aspiring to make content sourced from multiple different institutions available to a global audience must give much higher priority to issues of trust and language than content creators may have previously done. We found several means by which platform managers can build trust—most prominently, the transparent use of objective, validated content quality evaluation tools. Translation of content into appropriate languages was found to be a key enabler.

### Recommendations for Future Research

In addition to mapping existing literature, future empirical research should include systematic surveys of open access platform websites and interviews with their developers to provide deeper insight into their design, governance, and real-world implementation. There is a notable lack of longitudinal research evaluating the lasting impact of open access medical education platforms. Additionally, limited evidence exists on the structured integration of these resources into formal medical training programs. Sustainable models—particularly those detailing financial and operational strategies for the long-term maintenance of such platforms—are also rarely published.

### Implication of Findings

This review identifies from the literature actions that each stakeholder group can take to improve open access medical platforms and increase their impact. Recommendations are summarized in Table 4.

**Table 4:** Recommended actions for stakeholder groups.

Stakeholder group	Recommended actions
Learners	Use trusted curated compilations of resources Peer-to-peer recommendation of content
Training programs	Recommend specific content on curated compilations of resources to learners Require learner completion of certain content, with deadlines Develop a formal learner engagement strategy
Content designers and creators	Evaluate the quality of educational content using validated evaluation tools Share best practices among educators and content creators Recruit content creators from the same context as targeted learners and integrate learners’ experience into content Provide low-bandwidth and downloadable resources for learners with poor or expensive internet connectivity Map and categorize new and existing content to ensure new content fills gaps rather than duplicates existing content Integrate new resources into established curricula Combine self-directed cognitive learning with peer interaction and instructor presence Adhere to best practices in adult education, including interactive learning and learner-centered approaches, and the provision of immediate feedback Translate content into appropriate languages
Platform managers	Use Creative Commons licenses to clarify the legal status of content Host content on open access platforms and use free tools to reduce costs Enhance navigability and searchability with custom search tools and other tools Formally recognize the contribution of content creators

### Limitations

Only studies published in English were included, which may introduce language bias and limit the representation of perspectives from non-English–speaking regions. Furthermore, the majority of included studies originated from high-income countries, potentially narrowing the cultural and economic contexts considered. We recognize the possibility of funding bias influencing the evidence base. The exclusion of gray literature may also have led to the omission of relevant nonindexed reports or institutional documents. Changes in medical education technology are fast-paced, and the literature necessarily lags behind changes in learner behavior. Nevertheless, this review establishes a foundation for future research, implementation, and policy development in the field of open access medical education.

### Conclusions

Open access medical education has the potential to add significant value to preservice and in-service medical education worldwide. However, numerous challenges are preventing open access medical education platforms from achieving their potential, particularly a lack of content quality control, incompleteness of content, and the challenge of sustaining the necessary human and financial resources.

Ultimately, if open access medical education is to achieve its potential, much greater coordination and collaboration on a global scale is required—between learners, educational institutions, content creators, and platform managers.

## Appendix

Multimedia Appendix 1 PRISMA-ScR checklist.

## Abbreviations

FOAM: Free Open Access Medical Education
LMIC: low- and middle-income country
PRISMA-ScR: Preferred Reporting Items for Systematic Reviews and Meta-Analyses extension for Scoping Reviews
